# The Texas Medical College (Medical Department of the University of Texas)

**Published:** 1895-12

**Authors:** 


**The Texas Medical College** (Medical Department of the University of Texas).—There were 225 matriculants at the Texas Medical College at the beginning of the session, November 1 (ult). They were distributed as follows: First year matriculates, over 100; second year, over 50; third year, about 40, and about 30 in the two Pharmacy classes. Besides these there are several special students. The faculty expect by the time work is fairly begun (by now), the matriculants,—all *bona fide* students—will number not less than 235. This is a most encouraging showing for the fourth year of this young school.

It is gratifying to note the active support that is being given the home school by leading Texas physicians. While a few—clinging to their old *alma mater* (loving their State not less, but her more, perhaps), have sent their sons out of the State to get their education—some to old Tulane, some to old Jefferson—many are having their sons educated in Galveston; amongst the list we see, at a casual inspection, the names of W. F. Starly, Jr., son of W. F. Starly, Sr., of Tyler; E. B. Osborn, son of Dr. J. D. Osborn, of Cleburne, and J. C. Loggins, Jr., son Dr. J. C. Loggins, Sr., of Ennis, three of the best men and most popular physicians of Texas. That they will prove worthy sons of honored sires, we have no doubt. Their fathers' name will be a constant incentive to strive for excellence.

**The Students' Council** of the Texas Medical College, realizing the necessity of an "organ," or something in the nature of an escape pipe through which to vent their surplus wisdom and eloquence, have begun the publication of a pamphlet, and call it "The University Medical," leaving each reader to finish out the name—"journal," "organ," "pamphlet," "fog-horn," "blast furnace," or whatever each may consider most appropriate. We dub it the "University Medical Bantam"; it crows as lustily, in the first issue,—as a bantam, and struts worse, making as much noise as does its spunky little prototype upon the discovery of a morsel, in fact, is full of fuss and fight, as well as pluck, determination and high aims; altogether, is very creditable to the "boys." At the helm editorial, we recognize a familiar name: Osborn, Student E. B. Osborn, son of Dr. J. D. O., and grandson of the venerable and venerated Dr. T. C. Osborn, and seems to be a chip of the old block. We extend him and his bantam game cock the right hand of fellowship, and wish him, his colleagues and the venture, abundant success.

